# The Association Between Food Addiction and Weight Status in School-Age Children and Adolescents

**DOI:** 10.3389/fpsyt.2022.824234

**Published:** 2022-05-09

**Authors:** Dan Wang, Ke Huang, Erica Schulte, Wanying Zhou, Huiwen Li, Yuzheng Hu, Junfen Fu

**Affiliations:** ^1^National Clinical Research Center for Child Health, The Children’s Hospital, Zhejiang University School of Medicine, Hangzhou, China; ^2^Center for Weight, Eating, and Lifestyle Science, Drexel University, Philadelphia, PA, United States; ^3^Faculty of Education, University of Cambridge, Cambridge, United Kingdom; ^4^Department of Psychology and Behavioral Sciences, Zhejiang University, Hangzhou, China

**Keywords:** food addiction, overweight, obesity, children, adolescent

## Abstract

**Background:**

The association between food addiction (FA) and weight status in children and adolescents remains poorly understood. This study aimed to elucidate the association between FA and weight status using the validated Chinese version of the dimensional Yale Food Addiction Scale for Children 2.0 (dYFAS-C 2.0).

**Methods:**

Participants were enrolled from clinic visitors for regular physical check in a children’s hospital. The dYFAS-C 2.0 was translated into Chinese and validated using reliability and validity tests. The participants’ body mass index *Z* score (BMIZ) and waist-to-height ratio (WHtR) were used to characterize weight status. The FA severity was assessed using the translated dYFAS-C 2.0.

**Results:**

Among the 903 children and adolescents enrolled, 426 (47.2%) completed the survey [277 (65%) females and 149 (35%) males]. The Cronbach α of translated dYFAS-C 2.0 was 0.934, and confirmatory factor analysis indicated an acceptable model fit. FA correlated positively with BMIZ and WHtR in the whole sample after adjusting for the effect of gender (*p* < 0.001). Further analyses showed that the correlation remained significant in participants with BMIZ > 1 (*p* = 0.006) but not in those with BMIZ ≤ 1 (*p* = 0.220). However, the correlations between FA and WHtR were statistically significant in both participants with or without abdominal obesity (*p* < 0.05). The FA could explain 12.1 and 15.8% of variance in BMIZ and WHtR, respectively. The corresponding cutoff points of FA for excessive weight risk were 0.7 (BMIZ) and 0.4 (WHtR).

**Conclusion:**

The dYFAS-C 2.0 has good reliability and validity in the Chinese population. FA is associated with weight status characterized by BMIZ and WHtR, especially in participants with BMIZ > 1 and in those with abdominal obesity.

**Clinical Trial Registration:**

[www.chictr.org.cn], identifier [ChiCTR2100052239].

## Introduction

Overweight and obesity have affected at least 340 million children and adolescents globally, and this is becoming a public health issue (1), with increasing prevalence in developing countries compared to developed countries (2, 3). Overweight and obese children possess a greater risk of developing chronic diseases, including diabetes, and are more likely to experience poor mental health status (e.g., elevated symptoms of depression and anxiety) (4). Overweight and obesity have also been related to puberty (5, 6) and other developmental problems (7), including deficits in executive function (8).

Overconsumption of food is a leading cause of overweight and obesity (9). Interventions that target weight loss by restraining food consumption dominate the clinical practice, but the long-term effects are limited (10, 11). Little is known about why children with excessive weight eat beyond homeostatic and physical needs. Previous studies have demonstrated that children may not be fully aware of how much food they have consumed, and some children have demonstrated a loss of control over eating when consuming their favorite food (12, 13). Such addictive-like eating behaviors have been operationalized by the construct of food addiction (FA) characterized by compulsive eating, impaired control, tolerance/withdrawal, and risky use, similar to substance use disorders (14). Recently, an increasing number of studies have focused on the relationship between FA and overweight or obesity (Supplementary eFigure 1). However, most FA measurement tools were developed using American samples, and most studies on FA were conducted in developed countries (Supplementary eFigure 2), leaving the generalizability of FA to a population with different cultures/lifestyles undetermined. Moreover, the concept of “food addiction” is still controversial (15), indicating the need for more evidence. In addition, most previous researches have been conducted in adults, but few researches were conducted in children and adolescents. Therefore, FA remains poorly understood in pediatrics (Supplementary eFigure 3).

School-age children and adolescents are more impulsive than adults (16, 17) and are a risky population for substance use owing to the imbalanced development between reward and inhibitory control systems (18). The temptation to food, especially palatable food (e.g., pastries, fast food, and packaged snacks, such as chips), plays a vital role in forming strong eating motivation that extends beyond satiety (19). The high sugar and fat content in palatable food could disrupt the gut microbiota and influence brain neurotransmitter levels and functions (20), further modulating the hypothalamus to promote eating behavior (21). Besides, the intake of palatable food also changes the brain’s reward system by shifting the hunger–satiety continuum of perpetual hunger and weakened satiety (22, 23), triggering uncontrolled addictive-like behaviors that may contribute to overweight or obesity (24). Studies in adults showed that more severe FA was related to a higher BMI, and FA also had an association with other kinds of eating behavior (25). However, evidence concerning the association of FA and weight status is limited in children and adolescents, especially in developing countries without validated measurement tools.

To assess FA in children, Schiestl and Gearhardt developed the first version of Yale Food Addiction Scale for Children (YFAS-C) in accordance with criteria for substance use disorder in the *Diagnostic and Statistical Manual of Mental Disorders, Fourth Edition* (*DSM-IV*), to make a dichotomic diagnosis of FA (26). But in the fifth edition of *DSM* (*DSM−5*), both substance use disorder and behavioral addictions such as Internet gaming disorder were combined the symptoms into a single diagnosis (27). Many previous studies on addiction, including our own, also followed this single-dimensional framework (28, 29). Accordingly, the updated version of YFAS-C also followed *DSM-5* to adopt a dimensional scoring approach instead of dichotomic categorization of FA. However, this dimensional YFAS-C (dYFAS-C 2.0) has not been validated in other samples except the American population (30).

Therefore, this study aimed to validate the psychometric performance of the dCYFAS-C 2.0 in school-age children and adolescents in China and to explore the association between FA and weight status, especially in individuals with excessive weight status (i.e., overweight and obesity).

## Materials and Methods

### Translation and Preliminary Test of dYFAS-C 2.0

Schiestl and Gearhardt developed the dYFAS-C 2.0 in 2018 (30) based on criteria for substance use disorder in *DSM-5* (27) to characterize addictive-like food consumption behavior. In alignment with *DSM-5*, a dimensional approach rather than a categorical scoring method was adopted for the dYFAS-C 2.0 (30). With permission from the original authors, Beaton’s translation method (31) was used to translate this scale into Chinese (referred to as C-dCYFAS-C 2.0 thereafter) and was tested in Chinese samples. See Supplementary Methods for more details on the translation process (Supplementary Methods - “Translation Process”).

First, a pilot test was conducted with the preliminary version of the C-dCYFAS-C 2.0 using a convenience sample of 40 children and adolescents (aged 8–18 years) and their parents. The children and adolescents were asked if they could understand each item. If there was any confusion, the researcher would explain in detail and ask for the participants’ intended alternative expressions. Their parents were asked whether their children could understand similar questions. A total of 32 children/adolescents and their parents completed the preliminary version of C-dCYFAS-C 2.0. The other eight did not complete the interview and preliminary version of C-dCYFAS-C 2.0; their data were excluded from statistical analysis. Although the initial internal consistency was α = 0.965 for this preliminary version, minor modifications were made following the feedback from the pilot test participants to create the final version.

The modified C-dCYFAS 2.0 was sent to seven experts with multidisciplinary backgrounds to rate the content validity. The content validity index (CVI) of the scale was 0.77 (using the universal agreement method) and 0.97 (using the averaging method). The CVI results are detailed in Supplementary eFigure 4.

### Study Population

The cross-sectional study was conducted in a tertiary children’s hospital in Eastern China as a part of a series of scales/questionnaires translation and validation works. Participants were enrolled from clinic visitors who came to visit the Department of Growth and Development. The informed consent was obtained from each participant and his/her guardians before participation. The inclusion and exclusion criteria are detailed in Supplementary Material (**inclusion and exclusion criteria**). The Ethics Committee of Children’s Hospital of Zhejiang University, School of Medicine approved this project (approval no. 2020-IRB-179).

### Demographic Data

The demographic information, including children’s birthday, gender, grade, inhabitation, and family structure, was collected. Other measures are detailed below.

### Anthropometry Data

The trained researchers measured children’s height, weight, and waistline using standard tools. The measurement methods are detailed in Supplementary Material (*Anthropometry measure method*).

Body mass index (BMI) and BMI *Z* score (BMIZ) were calculated for each child/adolescent following his/her age and gender using the World Health Organization’s (WHO’s) Anthro Software (5–19 years) (32). Diagnosis for overweight and obesity was categorized in reference to both WHO’s standard (33) and Chinese Overweight and Obesity Screen standard (34) (supplementary: 6–18 years school-age children and adolescents overweight and obesity screening by BMI and gender).

Waist-to-height ratio (WHtR) was also calculated, and abdominal obesity was determined by WHtR ≥ 0.48 and WHtR ≥ 0.46 for boys and girls, respectively, according to the experts’ consensus on the definition and prevention of metabolic syndrome in Chinese children and adolescents (35).

### Addictive-Like Eating Behavioral Data

The C-dCYFAS-C 2.0 was used to measure the addictive-like eating behavior characterized by FA. This scale is a 35-item self-rated scale, with a 5-point Likert rating ranging from never (0) to always (4) for each item. Using the dimensional scoring approach, the C-dYFAS-C 2.0 assesses the severity of FA symptoms, with higher scores indicating more severe addictive-like eating symptom.

### Criterion-Related Validation Measures

The Chinese version of the Dutch Eating Behavior Questionnaire–Child version (DEBQ-C) and Chinese version of Children Eating Behavior Questionnaire (DEBQ) were used to explore the convergent validity of C-dCYFAS-C 2.0. The DEBQ-C (36), a widely used self-reported eating behavior measurements, was developed by Van Strien and colleagues. Zhao revised and validated the Chinese version of DEBQ-C used in this study (37). The higher scores indicate more severe problematic eating behavior (37). The Children Eating Behavior Questionnaire (CEBQ) is a proxy-reported measurement developed by Wardle et al. (38). The CEBQ is a 35-item measurement with eight dimensions (38). The higher scores indicate more severe problematic eating behavior (38). Xue translated and validated the Chinese version of CEBQ used in this study (39).

### Data Collection Process

**FIGURE 1:**
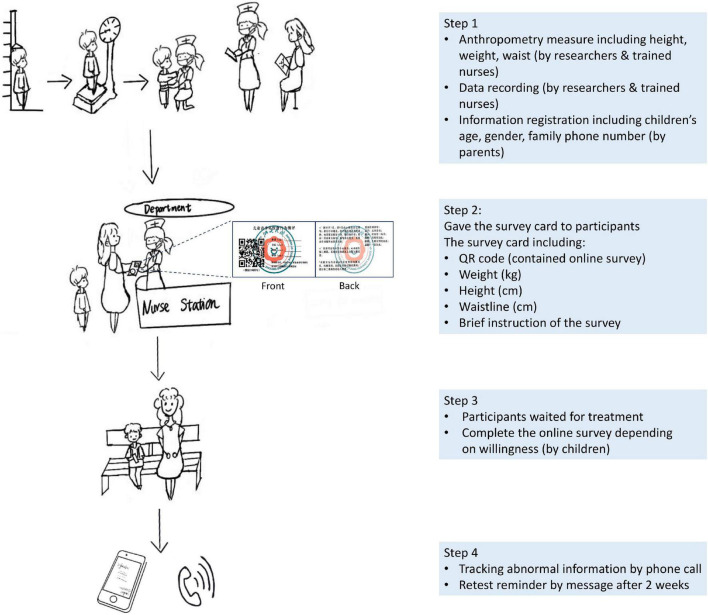
Data collection process.

The data collection process and materials are illustrated in Figure 1.

### Statistical Analysis

Participants’ characteristics were summarized using proportion (%), mean, and standard deviation or median and compared by two-sample independent *t-*test or Mann–Whitney *U*-test when appropriate. The internal consistency assessed the reliability using Cronbach α and McDonald’s ω, and test–retest reliability was assessed using the intraclass correlation coefficient (ICC). CVI, convergent validity, and confirmatory factor analysis (CFA) were used to assess the validity. Pearson or Spearman correlational analysis was used to analyze the relationship between FA and BMIZ/WHtR with gender as covariance. The between-subjects effects test was used to further examine the impact of gender on the relationship between FA and weight status. Subjects were divided into subgroups based on BMIZ/WHtR, and the comparison of FA scores among different subgroups was conducted using analysis of variance or Kruskal–Wallis *H*-test. Linear regression analysis was used to determine the contribution of FA to weight status, with adjusted *R*^2^ indicating the effect size. The receiver operating characteristic curve was used to further depict the relationship between FA and weight status, and the Youden index determined cutoff points. SPSS (version 23.0; IBM Corp., Armonk, NY, United States) and Jamovi (version 1.2.27.0) were used for statistical analyses. GraphPad Prism version 9.0 and R ggplot2 package were used for generating statistical figures.

## Results

### Participants Recruitment and Retention

**FIGURE 2:**
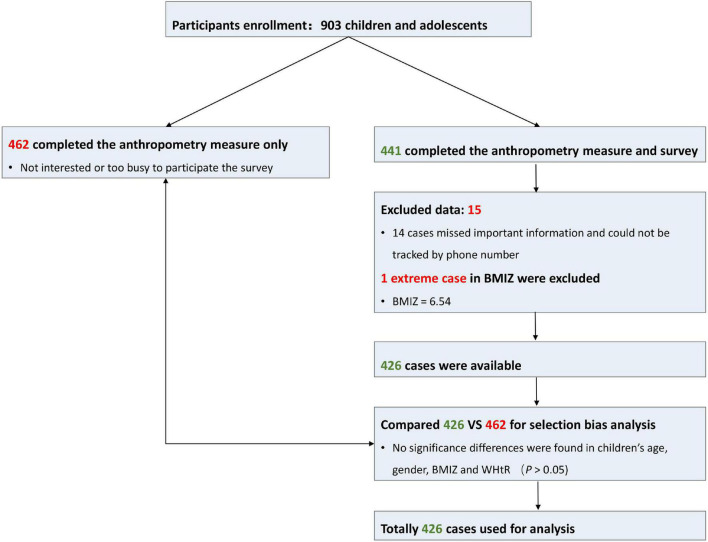
Participant recruitment and retention. BMIZ, body mass index *Z* score; WHtR, waist-to-height ratio.

Comparisons between participants of the survey sample and participants who did not take the survey were performed to test whether participants who completed the survey were biased in demographic and weight status characteristics or not. Insignificant differences were found between the two groups (Supplementary eTable 1). The participants’ recruitment and retention process are presented in Figure 2.

The mean age of the survey participants was 10.53 (SD = 1.68) years, ranging from 8.03 to 17.35 years. The boys had a higher excessive weight proportion than girls (37.6% vs. 20.9%, χ^2^ = 13.696, *p* < 0.01). Other descriptive statistics and comparisons are listed in Table 1. The prevalence of overweight/obesity was 26.8% by WHO and Chinese standards. There was no significant difference in the overweight and obesity diagnostic results using the two standards (κ = 0.917, *p* < 0.01). The WHO standard was used for the following analyses.

**TABLE 1:** The characters of participants and the association with weight status.

Item	*n* (%)	Different weight groups		*t/*χ^2^	*p*
		Normal/under weight	Overweight/ obesity		
Age	426	10.53 ± 1.71	10.55 ± 1.60	−0.111	0.911
Gender				13.696	<0.001
Girl	277 (65%)	219	58		
Boy	149 (35%)	93	56		
Inhibition				1.350	0.509
Cities	255 (59.9%)	191	64		
Suburb/small towns	119 (27.9%)	86	33		
Countryside	52 (12.2%)	35	17		
Family structure				0.053	0.818
Nuclear family (Live only with parents)	269 (63.1%)	196	73		
Stem family (Live with parents and grandparents)	157 (36.9%)	116	41		
School host				2.039	0.153
Yes	22 (5.2%)	19	3		
No	404 (94.8%)	293	111		
Grade				0.644	0.779
2–6 level	373 (87.6%)	273	100		
7–9 level	50 (11.7%)	36	14		
Other level	3 (0.7%)	3	0		

*The prevalence of overweight and obesity were 71 (16.7%) and 43 (10.1%), respectively, according to WHO’s standard and were 64 (15%) and 50 (11.7%), respectively, according to Overweight and Obesity Screen Standard in Chinese Children and Adolescents.*

### Reliability of C-dCYFAS-C 2.0

The Cronbach α and McDonald’s ω were 0.934 and 0.938, respectively, in the internal reliability analysis. The ICC was 0.761 (95% confidence interval [CI] = 0.534–0.882; *p* < 0.01) in the test–retest analysis.

### Validity of C-dCYFAS-C 2.0

The CVI results are displayed in Supplementary eFigure 4. The reliability values of DEBQ-C and DEBQ were 0.795 and 0.734, respectively. The C-dCYFAS-C 2.0 was correlated with DEBQ-C (*r* = 0.456, *p* < 0.001) and DEBQ (*r* = 0.336, *p* < 0.001), indicating a sound convergent validity.

The model fit indices of dCYFAS-C 2.0 (χ^2^/*df* = 4.04, goodness-of-fit index = 0.718, Tucker–Lewis index = 0.7, root mean square error of approximation = 0.084, and standardized root mean square residual = 0.067) suggested an acceptable model fit. The factor loading of each item is listed in Supplementary eTable 2.

### The Relationship Between Food Addiction and Body Mass Index *Z* Score/Waist-to-Height Ratio

In the present study, FA score was independent of age (*r* = 0.050, *p* = 0.299), but gender showed a marginally significant effect on FA (boys > girls: *t* = 1.866, *p* = 0.063). In later analyses on the relationship between FA and weight status, gender was included as a covariate.

**FIGURE 3:**
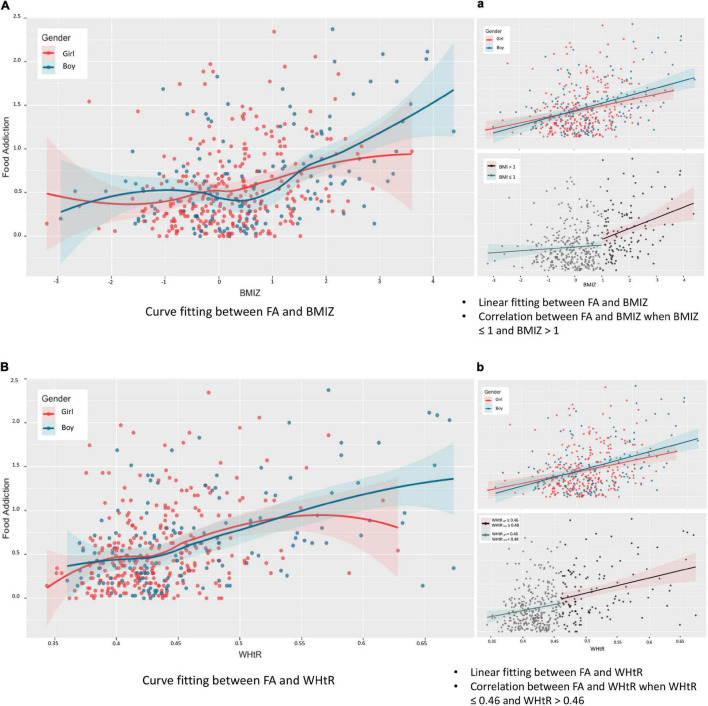
Relationship between food addiction and weight status. BMIZ, body mass index *Z* score; WHtR, waist-to-height ratio. Partial correlation analysis (controlling for gender): *r*_FA–BMIZ_ = 0.341 (95% CI = 0.248–0.435), *p* < 0.05. No interaction effect of gender was found between the association of FA and BMIZ (*F* = 0.829, *p* = 0.775). Spearman relationship: *r*_Boy_ = 0.362 (95% CI = 0.215–0.494), *p* < 0.05, *r*_Girl_ = 0.274 (95% CI = 0.164–0.387), *p* < 0.05 **(Aa)**. Partial correlation analysis (controlling for gender): *r*_FA–WHtR_ = 0.391 (95% CI = 0.288–0.484), *p* < 0.05. Interaction effect of gender was found between the association of FA and WHtR (*F* = 2.158, *p* < 0.05). Spearman relationship: *r*_Boy_ = 0.420 (95% CI = 0.283–0.541), *p* < 0.05, *r*_Girl_ = 0.306 (95% CI = 0.191–0.418), *p* < 0.05 **(Bb)**.

The FA was positively correlated with BMIZ (*r* = 0.341, *p* < 0.001; Figure 3A) and WHtR (*r* = 0.391, *p* < 0.001; Figure 3B) after controlling for the effect of gender. No significant difference was found between the strength of the two correlations.

When BMIZ ≤ 1, the correlation between FA and weight status was insignificant (*r*_FA–BMIZ_ = 0.070, *p* = 0.220), whereas when BMIZ > 1, the positive correlation between FA and weight status remained significant (*r*_FA–BMIZ_ = 0.258, *p* < 0.006), indicating the association between FA and weight status was prominent in the population clinically concerned as overweight/obesity based on BMIZ (Figure 3a).

When determining obesity using WHtR, the association between FA and weight status (WHtR) was significant in both participants without abdominal obesity (i.e., for boys with WHtR < 0.48 and girls with WHtR < 0.46, *r*_FA–WHtR_ = 0.131, *p* = 0.022) and participants with abdominal obesity (i.e., WHtR_boy_ ≥ 0.48 and WHtR_girl_ ≥ 0.46, *r*_FA–WHtR_ = 0.284, *p* = 0.002), indicating a stable association between FA and weight status characterized with WHtR (Figure 3b).

### The Comparisons of Addictive-Like Eating Behavior Between Subgroups

**FIGURE 4:**
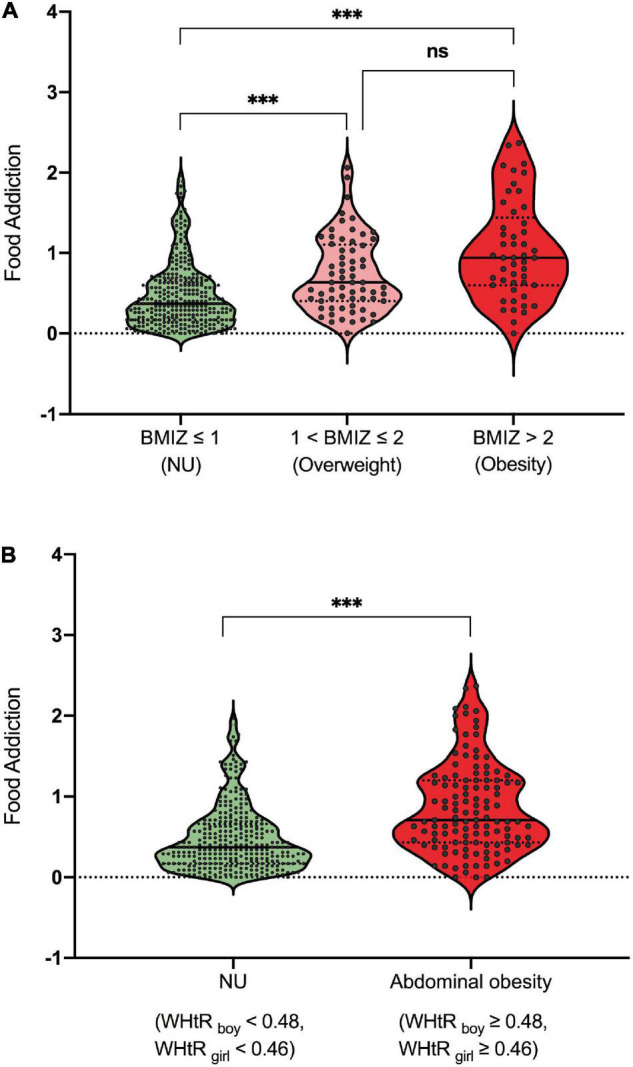
**(A,B)** Differences of addictive-like eating behavior across subgroups. ***p < 0.001. ns, not significant.

The participants were divided into three subgroups based on BMIZ, namely, NU (normal or underweight, BMIZ ≤ 1), overweight (1 < BMIZ ≤ 2), and obesity (BMIZ > 2). As shown in Figure 4A, the NU subgroup had lower FA scores than both overweight and obesity subgroups, but the latter two showed no difference. Participants were also divided into two subgroups according to their WHtR as NU (without abdominal obesity, WHtR_boy_ < 0.48, and WHtR_girl_ < 0.46) and abdominal obesity (WHtR_boy_ ≥ 0.48, WHtR_girl_ ≥ 0.46). As shown in Figure 4B, participants with overweight/obesity or abdominal obesity had more severe addictive-like eating behaviors than the NU ones. See Supplementary eTable 3 for detailed statistics.

### The Contribution of Food Addiction to Weight Status

The linear regression analysis showed that FA could explain 12.1% of the variance of BMIZ (standardized coefficients of β = 0.35, *t* = 7.697, *p* < 0.001, 95% CI = 0.635–1.071) and 15.8% of the variance of WHtR (standardized coefficients of β = 0.4, *t* = 8.985, *p* < 0.001, 95% CI = 0.039–0.060).

### The Food Addiction Cutoff Point for Normal-Weight/Underweight and Excessive Weight Groups

**FIGURE 5:**
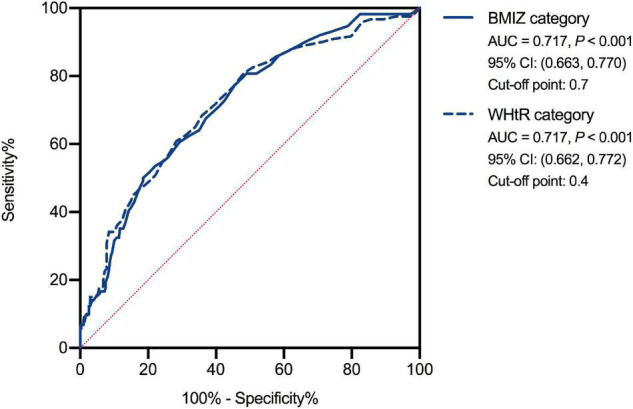
The receiver operating characteristic curve of food addiction for normal-weight/underweight and excessive weight groups. BMIZ, body mass index *Z* score; WHtR, waist-to-height ratio.

The AUCs of FA for discriminating participants with excessive weight from those with normal weight/underweight determined using BMIZ and WHtR were found similar (0.717, *p* < 0.001; Figure 5). Taking the Max Youden index as reference, the cutoff point using FA for normal weight/underweight and overweight/obesity was 0.7 (BMIZ). The cutoff point for normal weight/underweight and abdominal obesity was 0.4 (WHtR).

## Discussion

This study showed that the C-dCYFAS-C 2.0 has good internal and test–retest reliability (40), sound content (41), and convergent validity (42), with acceptable construct validity (43), indicating that it is a reliable measure of addictive-like eating behaviors in developing countries, such as China. The FA is positively associated with excessive weight status and could explain 12.1% of the variance of BMIZ as well as 15.8% of the variance of WHtR. The FA cutoff points to determine overweight/obesity were 0.4 and 0.7 when characterizing weight status using BMIZ and WHtR, respectively. These findings could help us understand better the association between addictive-like eating behaviors and overweight/obesity in children and adolescents, and they may have important implications for developing new intervention and prevention strategies for obesity.

In a previous study, a 17.3% prevalence of FA was found in 3,908 Iranian children and adolescents aged 7–13 years (44). An online cross-sectional study of 150 children in the United States reported 22.7% of the participants being diagnosed with FA. In contrast, in a validation study of the YFAS-Adult version in China, 9.2% of 950 female adolescents were diagnosed with FA (45). A cross-sectional study on a large sample (*n* = 1,144) using general Russian adolescents reported a 4.5% prevalence of FA (46). In contrast, a clinical study showed that the FA diagnosis was 38% in 50 adolescents seeking for weight-loss treatment (47). In a recent meta-analysis, the average FA prevalence was 15% (95% CI = 11–19%) in the pediatric population, with a higher prevalence of 19% in children and adolescents with overweight or obesity (48). In short, these studies showed that FA is prevalent in children and adolescents, but the prevalence varies from 4.5 to 38% across studies, dependent on countries and sample characteristics. As our participants were recruited from clinical visitors, the frequency of overweight/obesity was higher than the general populations reported in previous studies (e.g., 9.4% in 46). Similarly, the overall severity of FA may also be higher in our sample than a random general sample. But because the new version of YFAS-C characterizes FA as a continuum instead of categorical diagnosis as its previous version does, a direct comparison of the FA prevalence between the current study and previous findings is difficult because of the change in the scoring approach.

In this study, addictive-like eating behaviors were found to be positively correlated with weight status, even when considering the covariance of gender. A previous study showed that the frequency of detection of FA in children and adolescents was dependent on age (46). But in the present study, the correlation between FA score and age was not significant. Consistent with our result, scores on the dYFAS−C 2.0 were not different among 13–16−year−old participants from a community sample in the original work developing the scale (30). Such discrepancy might be attributed to the change of the dYFAS-C 2.0 diagnostic criteria. Compared with children with normal weight/underweight, children with excessive weight status had higher FA scores. This finding aligned with the previously reported results in adults (30, 49).

The association between FA and excessive weight status observed in this study may be attributed to elevated reward responsiveness toward palatable and/or ultraprocessed foods reported in previous studies (23, 24). Children and adolescents with excessive weight showed enhanced nucleus accumbens responsivity (50) when facing food cues and had poor inhibitory control in general (51). Consequently, these individuals were vulnerable to overeating. In addition, the dysfunction of the D_2_ receptor (52) may reflect an imbalance in the reward and control systems in addicted individuals. Such imbalance could lead to addictive-like eating behavior and contribute to the development of overweight and obesity (53). As the reward system develops in a curvilinear trajectory with a fast mature rate during late childhood and a developmental peak during adolescence (54), children and adolescents are at a higher risk of developing problematic behavior, including addictive-like eating. Therefore, exploring the underlying neural mechanism of FA in children and adolescents with excessive weight status may help identify risky neuropsychological factors of obesity, and it is also essential for developing targeted and effective interventions with long-lasting therapeutic gains.

Moreover, the positive correlation between FA and BMIZ could only be found in participants with BMIZ > 1, not those with BMIZ ≤ 1, indicating that FA was associated with overweight and obesity. In contrast to such a dichotomic relationship between FA and BMIZ, the FA related positively to WHtR regardless of weight status, indicating a linear dose–effect relationship between FA and abdominal obesity. Furthermore, the FA could explain 15.8% of the variance of WHtR, which was higher than that of BMIZ of 12.1%, indicating a possible closer relationship between FA and fat distribution. BMIZ was calculated from height and total body weight without considering the fat distribution. Some children or adolescents possessing higher BMIZ may be due to higher muscle possession (55), which signifies a strong and healthy body rather than excessive fat. In contrast, WHtR is related to belly fat amount that is related to an unhealthy condition. Therefore, WHtR is more sensitive in predicting the risk of metabolic and cardiovascular diseases (55). It seemed that FA has a tighter relationship with fat distribution. Future studies should consider body fat composition rather than focus on BMI only.

Furthermore, this study determined the cutoff points of FA between the normal-weight/underweight group and excessive weight groups. The cutoff points were 0.7 when considering weight status from the BMIZ perspective and 0.4 when using WHtR. These threshold scores could be used as references to categorize individuals as high/low risk of obesity from FA, making FA a potential measurement for overweight/obesity prevention and treatment response index. But a longitudinal cohort design is needed to validate further the causal relationship between FA and overweight/obesity. Moreover, as FA concerns behaviors similar to those in substance abuse, some effective interventions for drug addiction or other addictive behaviors, such as cognitive behavior therapy (56) and mindful training (57), could also be used to treat addictive-like eating behavior.

These results provided evidence of a significant correlation between FA and weight status, which indicated that FA should be considered when treating childhood overweight and obesity, especially in individuals with compulsive eating behavior. In addition, body composition, such as fat content, is worth giving attention in obesity research rather than considering weight only. The threshold of FA scores could be used for high FA risk screening and used as references for exposure subgroup categorization when conducting a cohort study.

### Strengths and Limitations

A key strength of this investigation is that the association between FA and overweight/obesity in school-aged children and adolescents in a developing country was established. In contrast to self-reported anthropometry data in previous studies, this study measured all anthropometry indices using standard tools and methods, conducted by trained nurses and researchers, which should have increased its reliability and validation.

Results from this single-center study may not generalize to children and adolescents from other countries. However, with the increasing prevalence of overweight and obesity, especially in developing countries, the evidence from China may give an insight into the association between FA and excessive weight status. Because of the inherent limitation of the cross-sectional design, this study could not determine the causal relationship between FA and weight status, and the population enrolled using the simple convenience sampling method from the hospital may limit the generalization of findings. Longitudinal designs are needed to provide a more robust evidence on the relationship between FA and weight status, especially their causal relationship. Also, large individual variances in Figures 3, 4 were observed. The fact that participants with high FA may not have a higher BMIZ or WHtR indicated that, in addition to FA, there are other factors accounting for excessive weight. Thus, further exploration of these factors is needed. Inversely, similar to findings of substance abuse, showing that the amount of substance intake may not account for severe behavior problems (58), excessive weight may not be related to addictive-like behaviors in some cases. Lastly, compensatory behaviors such as exercise (59) that were not measured in this study might influence the correlation between FA and weight status.

## Conclusion

The C-dCFAS 2.0-C had good reliability and validity in Chinese children and adolescents. These findings uncover the association between FA and weight status, especially in individuals with overweight and obesity. These findings could help us to understand better how addictive-like eating behaviors contribute to overweight/obesity in children and adolescents and may illuminate new intervention and prevention strategies for obesity.

## Data Availability Statement

The raw data supporting the conclusions of this article will be made available by the authors, without undue reservation.

## Ethics Statement

The studies involving human participants were reviewed and approved by the Ethics Committee of Children’s Hospital of Zhejiang University. Written informed consent to participate in this study was provided by the participants’ legal guardian/next of kin.

## Author Contributions

JF and YH made the contribution to conception. DW and KH made the contribution to the study design and data analysis. HL and DW made the contribution to acquisition of data, analysis and interpretation of data. DW drafted the manuscript. ES and WZ revising the manuscript critically for important intellectual content. All authors gave the final approval to the submitted version.

## Conflict of Interest

The authors declare that the research was conducted in the absence of any commercial or financial relationships that could be construed as a potential conflict of interest.

## Publisher’s Note

All claims expressed in this article are solely those of the authors and do not necessarily represent those of their affiliated organizations, or those of the publisher, the editors and the reviewers. Any product that may be evaluated in this article, or claim that may be made by its manufacturer, is not guaranteed or endorsed by the publisher.

## References

[1] Available online at: https://www.who.int/news-room/fact-sheets/detail/obesity-and-overweight

[2] Ng M Fleming T Robinson M Thomson B Graetz N Margono C. Global, regional, and national prevalence of overweight and obesity in children and adults during 1980-2013: a systematic analysis for the Global Burden of Disease Study 2013. *Lancet (London, England).* (2014) 384:766–81. 10.1016/S0140-6736(14)60460-8 24880830 PMC4624264

[3] Garrido-Miguel M Cavero-Redondo I Álvarez-Bueno C Rodríguez-Artalejo F Moreno LA Ruiz JR. Prevalence and trends of overweight and obesity in European children from 1999 to 2016: a systematic review and meta-analysis. *JAMA Pediatr.* (2019) 173:1–13. 10.1001/jamapediatrics.2019.2430 31381031 PMC6686782

[4] Jebeile H Gow ML Baur LA Garnett SP Paxton SJ Lister NB. Association of pediatric obesity treatment, including a dietary component, with change in depression and anxiety: a systematic review and meta-analysis. *JAMA Pediatrics.* (2019) 173:e192841–192841. 10.1001/jamapediatrics.2019.2841 31524933 PMC6749546

[5] Nokoff N Thurston J Hilkin A Pyle L Zeitler PS Nadeau KJ. Sex differences in effects of obesity on reproductive hormones and glucose metabolism in early puberty. *J Clin Endocrinol Metab.* (2019) 104:4390–7. 10.1210/jc.2018-02747 30985874 PMC6736047

[6] Press M. *Obesity Speeds Up The Start of Puberty in Boys, Study Finds.* (2019). Available online at: https://medicalxpress.com/news/2019-03-obesity-puberty-boys.html (accessed 2021).

[7] Prenticea MP. The double burden of malnutrition in countries passing through the economic transition. *Ann Nutr Metab.* (2018) 72 (Suppl 3):47–54. 10.1159/000487383 29635233

[8] Laurent JS Watts R Adise S Allgaier N Chaarani B Garavan H. Associations among body mass index, cortical thickness, and executive function in children. *JAMA Pediatr.* (2020) 174:170–7. 10.1001/jamapediatrics.2019.4708 31816020 PMC6902097

[9] Liang J Matheson BE Rhee IE Peterson CB Rydell S Boutelle IN. Parental control and overconsumption of snack foods in overweight and obese children. *Appetite.* (2016) 100:181–8. 10.1016/j.appet.2016.02.030 26911259 PMC4799726

[10] Thivel D Julian V Miguet M Pereira B Beaulieu K Finlayson G. Introducing eccentric cycling during a multidisciplinary weight loss intervention might prevent adolescents with obesity from increasing their food intake: the Textoo study. *Physiol Behav.* (2020) 214:1–28. 10.1016/j.physbeh.2019.112744 31765664

[11] Geiker NRW Astrup A Hjorth MF Sjodin A Pijls L Markus CR. Does stress influence sleep patterns, food intake, weight gain, abdominal obesity and weight loss interventions and vice versa? *Obes Rev.* (2018) 19:81–97. 10.1111/obr.12603 28849612

[12] Booth C Spronk D Grol M Fox E. Uncontrolled eating in adolescents: the role of impulsivity and automatic approach bias for food. *Appetite.* (2018) 120:636–43. 10.1016/j.appet.2017.10.024 29066344 PMC5689136

[13] Cohen DA Farley TA. Peer reviewed: eating as an automatic behavior. *Prevent Chronic Dis.* (2008) 5:1–7. PMC2248777 18082012

[14] Gordon EL Ariel-Donges AH Bauman V Merlo LJ. What is the evidence for “Food Addiction?”. A systematic review. *Nutrients.* (2018) 10:477. 10.3390/nu10040477 29649120 PMC5946262

[15] Corsica JA Pelchat ML. Food addiction: true or false? *Curr Opin Gastroenterol.* (2010) 26:165–9. 10.1097/MOG.0b013e328336528d 20042860

[16] Harvanko AM Strickland JC Slone SA Shelton BJ Reynolds BA. Dimensions of impulsive behavior: predicting contingency management treatment outcomes for adolescent smokers. *Addict Behav.* (2019) 90:334–40. 10.1016/j.addbeh.2018.11.031 30508743 PMC6425739

[17] Argyriou E Um M Carron C Cyders MA. Age and impulsive behavior in drug addiction: a review of past research and future directions. *Pharmacol Biochem Behav.* (2018) 164:106–17. 10.1016/j.pbb.2017.07.013 28778737 PMC5797988

[18] Centers for Disease Control and Prevention [CDC]. Youth risk behavior Surveillance-United States, 2019. *Morbidity Mortality Weekly Report MMWR* (2020) 69:1–88.31917782

[19] Miller AL Riley H Domoff SE Gearhardt AN Sturza J Kaciroti N. Weight status moderates stress-eating in the absence of hunger associations in children. *Appetite.* (2019) 136:184–92. 10.1016/j.appet.2019.02.005 30771403 PMC6755672

[20] Guo Y Zhu X Zeng M Qi L Tang X Wang D. A diet high in sugar and fat influences neurotransmitter metabolism and then affects brain function by altering the gut microbiota. *Transl Psychiatry.* (2021) 11:328. 10.1038/s41398-021-01443-2 34045460 PMC8160265

[21] Kleinman ER. *Pediatric Nutrition.* Itasca, IL: American Academy of Pediatrics (2014).

[22] Bennett C Burrows T Pursey K Poudel G Ng KW Nguo K. Neural responses to food cues in middle to older aged adults: a scoping review of fMRI studies. *Nutr Dietetics J Dietitians Assoc Australia.* (2020) 78:343–64. 10.1111/1747-0080.12644 33191542

[23] Olszewski PK Wood EL Klockars A Levine AS. Excessive consumption of sugar: an insatiable drive for reward. *Curr Nutr Rep.* (2019) 2019:120–8. 10.1007/s13668-019-0270-5 30945139

[24] Shearrer GE Stice E Burger KS. Adolescents at high risk of obesity show greater striatal response to increased sugar content in milkshakes. *Am J Clin Nutr.* (2018) 107:859–66. 10.1093/ajcn/nqy050 29771283 PMC6037118

[25] Gearhardt AN Corbin WR Brownell KD. Development of the yale food addiction scale Version 2.0. *Psychol Addict Behav J Soc Psychol Addict Behav.* (2016) 30:113–21. 10.1037/adb0000136 26866783

[26] Gearhardt AN Roberto CA Seamans MJ Corbin WR Brownell KD. Preliminary validation of the yale food addiction scale for children. *Eat Behav.* (2013) 14:508–12. 10.1016/j.eatbeh.2013.07.002 24183146 PMC3817415

[27] American Psychiatric Association [APA]. *Diagnostic and Statistical Manual of Mental Disorders.* 5th ed. Washington DC: Amercian Psychiatric Publishing (2013).

[28] Hu YZ Salmeron BJ Gu H Stein EA Yang YH. Impaired Functional Connectivity within and between frontostriatal circuits and its association with compulsive drug use and trait impulsivity in cocaine addiction. *JAMA Psychiatry.* (2015) 72:584–92. 10.1001/jamapsychiatry.2015.1 25853901

[29] Zhang J Zhou H Geng F Song X Hu Y. Internet gaming disorder increases mind-wandering in young adults. *Front Psychol.* (2020) 11:619072. 10.3389/fpsyg.2020.619072 33584453 PMC7876259

[30] Schiestl ET Gearhardt AN. Preliminary validation of the yale food addiction scale for children 2.0: a dimensional approach to scoring. *Eur Eat Disord Rev.* (2018) 26:605–17. 10.1002/erv.2648 30334311 PMC6231957

[31] Beaton DE Bombardier C Guillemin F Ferraz MB. Guidelines for the process of cross-cultural adaptation of self-report measures. *Spine.* (2000) 25:3186–91. 10.1097/00007632-200012150-00014 11124735

[32] Available online at: https://www.who.int/tools/growth-reference-data-for-5to19-years/application-tools (accessed 2021).

[33] Available online at: https://www.who.int/tools/growth-reference-data-for-5to19-years/indicators/bmi-for-age (accessed 2021).

[34] American Psychiatric Association [APA]. *Screening for Overweight and Obesity Among School-Age Children and Adolescents: Domestic Industry Standard-Industry Standard-Health CN-WS.* Washington DC: Amercian Psychiatric Publishing (2013).

[35] Fu J Liang L. Definition and prevention of metabolic syndrome in Chinese children and adolescents. *Chin J Pediatr.* (2012) 50:420–2. 10.3760/cma.j.issn.0253-9624.2018.11.007 22931932

[36] Van Strien T Oosterveld P. The children’s DEBQ for assessment of restrained, emotional, and external eating in 7- to 12-year-old children. *Int J Eat Disord.* (2008) 41:72–81. 10.1002/eat.20424 17634965

[37] Zhao Y. *Application of Revised The Dutch Eating Behavior Questionnaire Child-Version for Chinese Children.* Wuhan: Wuhan Sports University (2018).

[38] Wardle J Guthrie CA Sanderson S Rapoport L. Development of the Children’s eating behaviour questionnaire. *J Child Psychol Psychiatry All Disciplines.* (2001) 42:963–70. 10.1111/1469-7610.00792 11693591

[39] Xue K. *Research on the Relationship and Risk Factors of Childhood Obesity and Appetite.* Shanghai: Fudan University (2012).

[40] Revelle W Zinbarg RE. Coefficients alpha, beta, omega, and the glb: comments on Sijtsma. *Psychometrika.* (2009) 74:145–54. 10.1007/s11336-008-9102-z

[41] Polit DF Beck CT Owen SV. Is the CVI an acceptable indicator of content validity? Appraisal and recommendations. *Res Nurs Health.* (2007) 30:459–67. 10.1002/nur.20199 17654487

[42] Devon HA Block ME Moyle-Wright P Ernst DM Hayden SJ Lazzara DJ. A psychometric toolbox for testing validity and reliability. *J Nurs Scholarship.* (2007) 39:155–64. 10.1111/j.1547-5069.2007.00161.x 17535316

[43] Ji-Qian F. *Statistical Methods for Biomedical Research.* Beijing: Higher Education Press (2007).

[44] Naghashpour M. Prevalence of food addiction among Iranian children and adolescents: associations with sociodemographic and anthropometric indices. *Med J Islamic Repub Iran.* (2018) 32:1–10. 10.14196/mjiri.32.8 30159259 PMC6108267

[45] Chen G Tang ZL Guo GP Liu XQ Xiao SY. The Chinese version of the Yale Food Addiction Scale: an examination of its validation in a sample of female adolescents. *Eat Behav.* (2015) 18:97–102. 10.1016/j.eatbeh.2015.05.002 26026613

[46] Borisenkov MF Tserne TA Bakutova LA. Food addiction in Russian adolescents: associations with age, sex, weight, and depression. *Eur Eat Disord Rev J Eat Disord Assoc.* (2018) 26:671–6. 10.1002/erv.2644 30318852

[47] Meule A Nill T Kübler A. Food addiction in overweight and obese adolescents seeking weight-loss treatment: food addiction in obese adolescents. *Eur Eat Disord Rev.* (2015) 23:193–8. 10.1002/erv.2355 25778000

[48] Yekaninejad MS Badrooj N Vosoughi F Lin C-Y Potenza MN Pakpour AH. Prevalence of food addiction in children and adolescents: a systematic review and meta-analysis. *Obes Rev.* (2021) 22:1–12. 10.1111/obr.13183 33403795 PMC8244111

[49] Khine MT Ota A Gearhardt AN Fujisawa A Morita M Minagawa A. Validation of the Japanese version of the yale food addiction scale 2.0 (J-YFAS 2.0). *Nutrients.* (2019) 11:687. 10.3390/nu11030687 30909486 PMC6471687

[50] Ferrario CR. Why did I eat that? Contributions of individual differences in incentive motivation and nucleus accumbens plasticity to obesity. *Physiol Behav.* (2020) 227:113114. 10.1016/j.physbeh.2020.113114 32777311 PMC8668087

[51] Hardee JE Phaneuf C Cope L Zucker R Gearhardt A Heitzeg M. Neural correlates of inhibitory control in youth with symptoms of food addiction. *Appetite.* (2020) 148:104578. 10.1016/j.appet.2019.104578 31904390 PMC7024015

[52] Kim B Yoon S Nakajima R Lee HJ Lim HJ Lee Y-K. Dopamine D2 receptor-mediated circuit from the central amygdala to the bed nucleus of the stria terminalis regulates impulsive behavior. *Proc Natl Acad Sci U S A.* (2018) 115:E10730–9. 10.1073/pnas.1811664115 30348762 PMC6233075

[53] Baik J. Dopamine signaling in food addiction: role of dopamine D2 receptors. *BMB Rep.* (2013) 46:519–26. 10.5483/bmbrep.2013.46.11.207 24238362 PMC4133846

[54] Shulman E Smith A Silva K Icenogle G Duell N Chein J. The dual systems model: review, reappraisal, and reaffirmation. *Dev Cogn Neurosci.* (2015) 17:1–16. 10.1016/j.dcn.2015.12.010 26774291 PMC6990093

[55] Alalwan TA. Phenotypes of sarcopenic obesity: exploring the effects on peri-muscular fat, the obesity paradox, hormone-related responses and the clinical implications. *Geriatrics (Basel, Switzerland).* (2020) 5:1–15. 10.3390/geriatrics5010008 32075166 PMC7151126

[56] Liddle HA Dakof GA Turner RM Henderson CE Greenbaum PE. Treating adolescent drug abuse: a randomized trial comparing multidimensional family therapy and cognitive behavior therapy. *Addiction.* (2008) 103:1660–70. 10.1111/j.1360-0443.2008.02274.x 18705691

[57] Vanzhula IA Levinson CA. Mindfulness in the treatment of eating disorders: theoretical rationale and hypothesized mechanisms of action. *Mindfulness.* (2020) 11:1–15. 10.1080/10640266.2011.547136 21181575

[58] Hu YZ Salmeron BJ Krasnova IN Gu H Lu HB Bonci A. Compulsive drug use is associated with imbalance of orbitofrontal- and prelimbic-striatal circuits in punishment-resistant individuals. *Proc Natl Acad Sci U S A.* (2019) 116:9066–71. 10.1073/pnas.1819978116 30988198 PMC6500166

[59] Gearhardt AN Schulte EM. Is food addictive? A review of the science. *Annu Rev Nutr.* (2021) 41:1–24. 10.1146/annurev-nutr-110420-111710 34152831

