# Supplementary material for: The Association Between Food Addiction and Weight Status in School-Age Children and Adolescents
Source: Front Psychiatry. 2022 May 9;13:824234. doi: 10.3389/fpsyt.2022.824234 (PMC9125319; doi:10.3389/fpsyt.2022.824234)
Supplement: Supplementary file 1 [file Data_Sheet_1.doc]

**The Association between Food Addiction and Weight Status in School-age Children and Adolescents**

Dan Wang, Ke Huang, Erica Schulte, Wanying Zhou, Huiwen Li, Yuzheng Hu, Junfen Fu

**Online Supplemental Materials**

**Introduction**

**The Search Strategy of Bibliometric Analysis for Introduction**

(food addict*) AND (child* OR kid* OR adolescent* OR teenager* OR school age) AND (obesity OR fat OR overweight OR adiposity)

Web of Science Core Collection (1900-2020)

220 results

The results were analyzed by CiteSpace 5.6 R4.


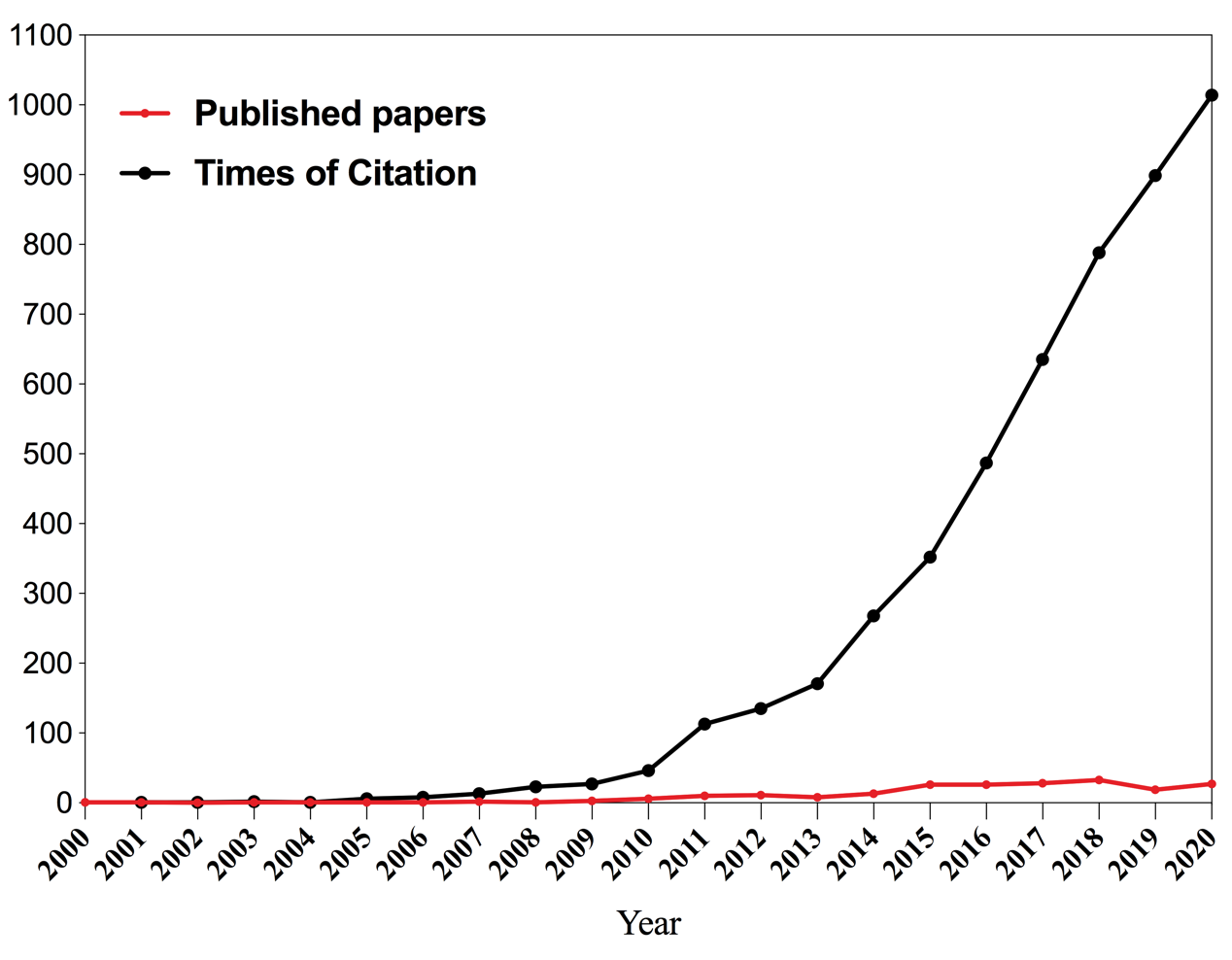


**eFigure 1 The Tendency of Research Interest of Food Addiction in Children and Adolescents**

Note: The figure showed an increasing interest in the studies of relationship between food addiction (FA) and overweight/obesity in children and adolescents.


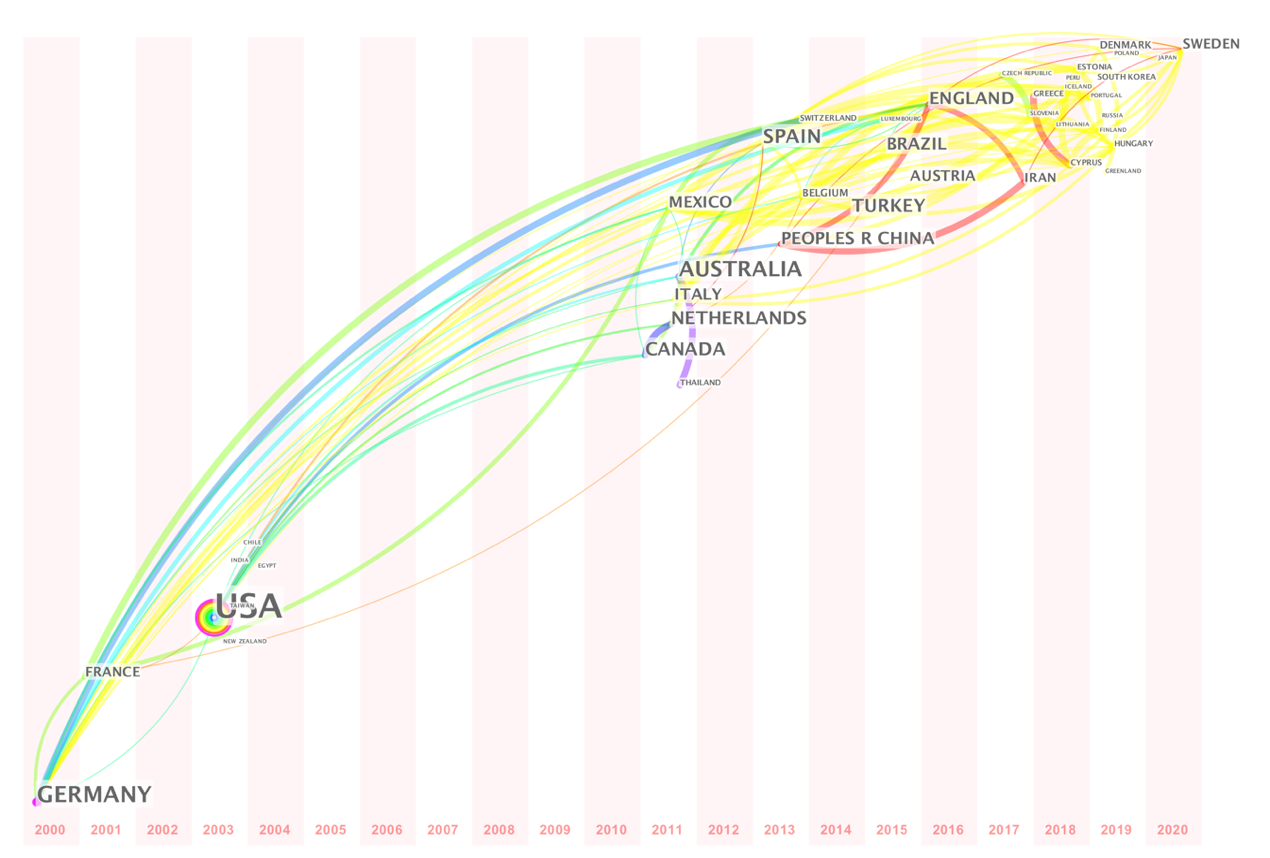


**eFigure 2 The Countries and Areas Evolved in Food Addiction Research in Children and Adolescents**

Note: The figure showed more and more countries and areas evolved in the stuides of FA and overweight/obesity in children and adolescents over time, especially in recent 10 years. The USA and other developed countries account for most studies. More cooperation was also found between countries and areas.


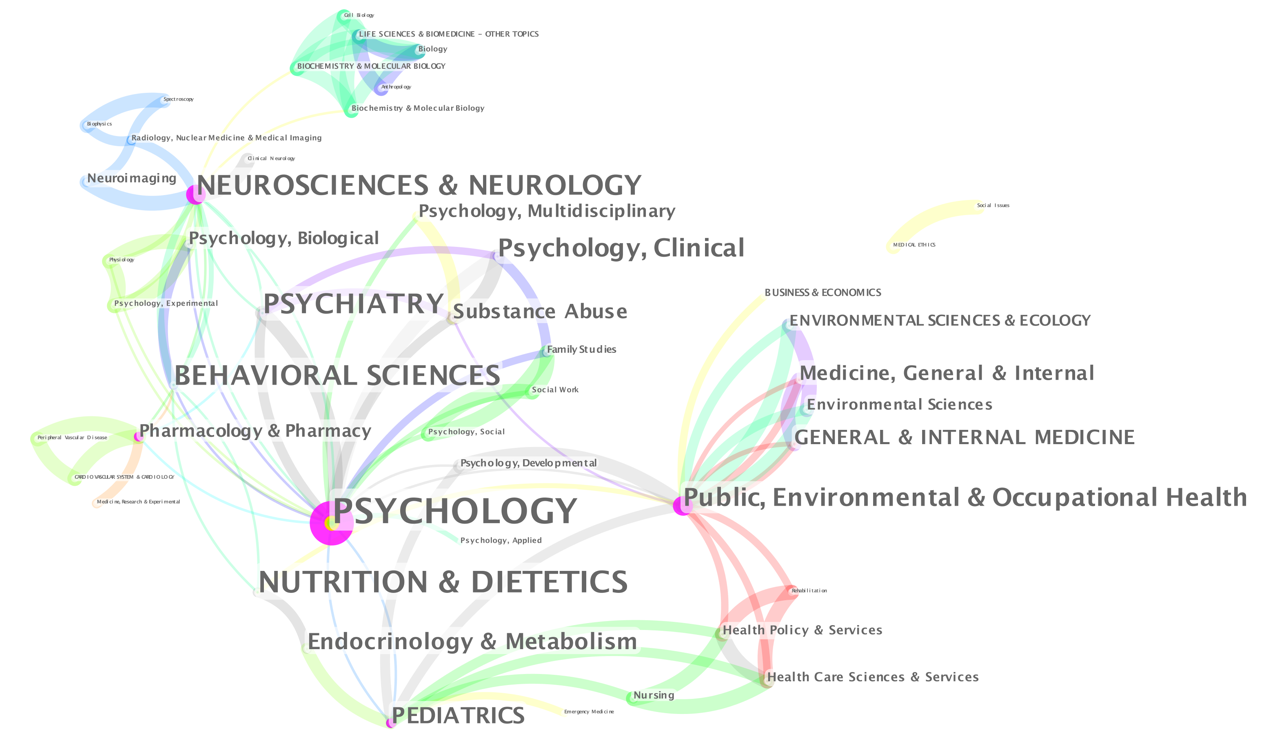


**eFigure 3 The Disciplines Distribution of the Research of Food Addiction in Children and Adolescents**

Note: Although studies concerned FA and overweight/obesity in children and adolescents were carried out in multi-discipline, related studies were still sparse in pediatrics.

**Methods**

**Translation Process**

Two researchers with Master’s degrees in Pediatrics (T1) and Nursing (T2) who were proficient in English and simplified Chinese forward translated the scales from English to simplified Chinese independently and then developed a consensus version through multiple discussions.

Another two translators with Master’s degrees in English (T3) and Psychology (Bilingual) (T4) who were blinded to the original English version independently back translated the Chinese version into English. The first author compared the back translation of dimensional Yale Food Addiction Scale for Children 2.0 (dYFAS-C 2.0) with the original English version in equivalence conceptually and semantically. The translation errors and inaccurate translations were reported back to T1 and T2 for re-translation. After that, a new consensus Chinese version was sent to T3 and T4 for re-work. After several rounds of translations and back-translations, the unsolved ambiguous expressions were recorded and sent to the authors of the original version for clarity. After receiving the feedbacks, the translators corrected the misunderstanding of expressions with the help of a native English speaker who has studied in China for 7 years. The final back translation was sent back to the original author of the dYFAS-C 2.0. The preliminary Chinese version of dYFAS-C 2.0 (C-dYFAS-C 2.0) was ready.

After pilot study, the modified C-dYFAS-C 2.0 was sent to 7 experts with multidisciplinary backgrounds to rate the content validity. The content validity index (CVI) results were detailed in eFigure 4.

**
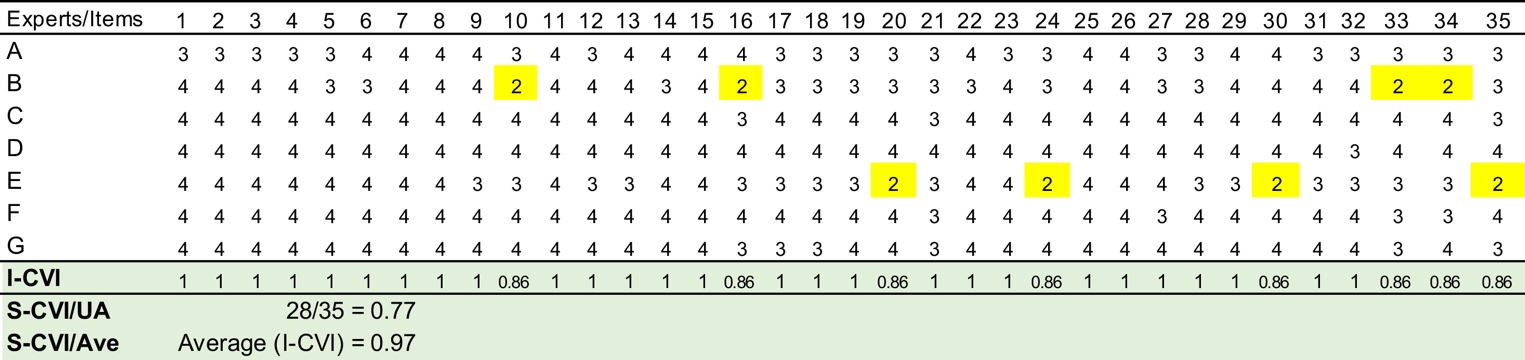
**

**eFigure4 The Results of CVI of C-dYFAS-C 2.0**

**Inclusion and exclusion criteria**

The whole project comprised 4 translated measurements concerning children’s eating behaviors. The participants (≥ 8 years) came to hospital for health check-up were enrolled. Children with diabetes, hyperthyroidism, heart disease, severe liver and kidney dysfunction, malignant tumor, autoimmune disease, hematological disease, and severe infection were excluded, as well as those with mental psychiatry problems and mental retardation. That information could be acquired through their medical visit records or enquiring parents with usage permission.

Parents with major negative events in recent 3 months, such as divorce, diagnosed with cancer, were also excluded, as the family event could affect the children’s behaviors.

In this study, we only illustrate the related work of dYFAS-C 2.0 translation and validation, as well as the association between food addiction and weight status.

**Anthropometry Measure Method**

**Take reference from Anthropometric measurements method in health surveillance**

Health industry standard of People’s Republic of China WS/T 424-2013

Released by National Health Commission of the People’s Republic of China

**Measurement tools**

Height: Seca213; Weight: Seca799; Waist: Seca201.（Seca incorporation, Germany）

Weight is measured after taking off shoes, coats and other heavy clothes, and the reading is accurate to 0.1kg.

Height and waistline is accurate to 0.1cm.


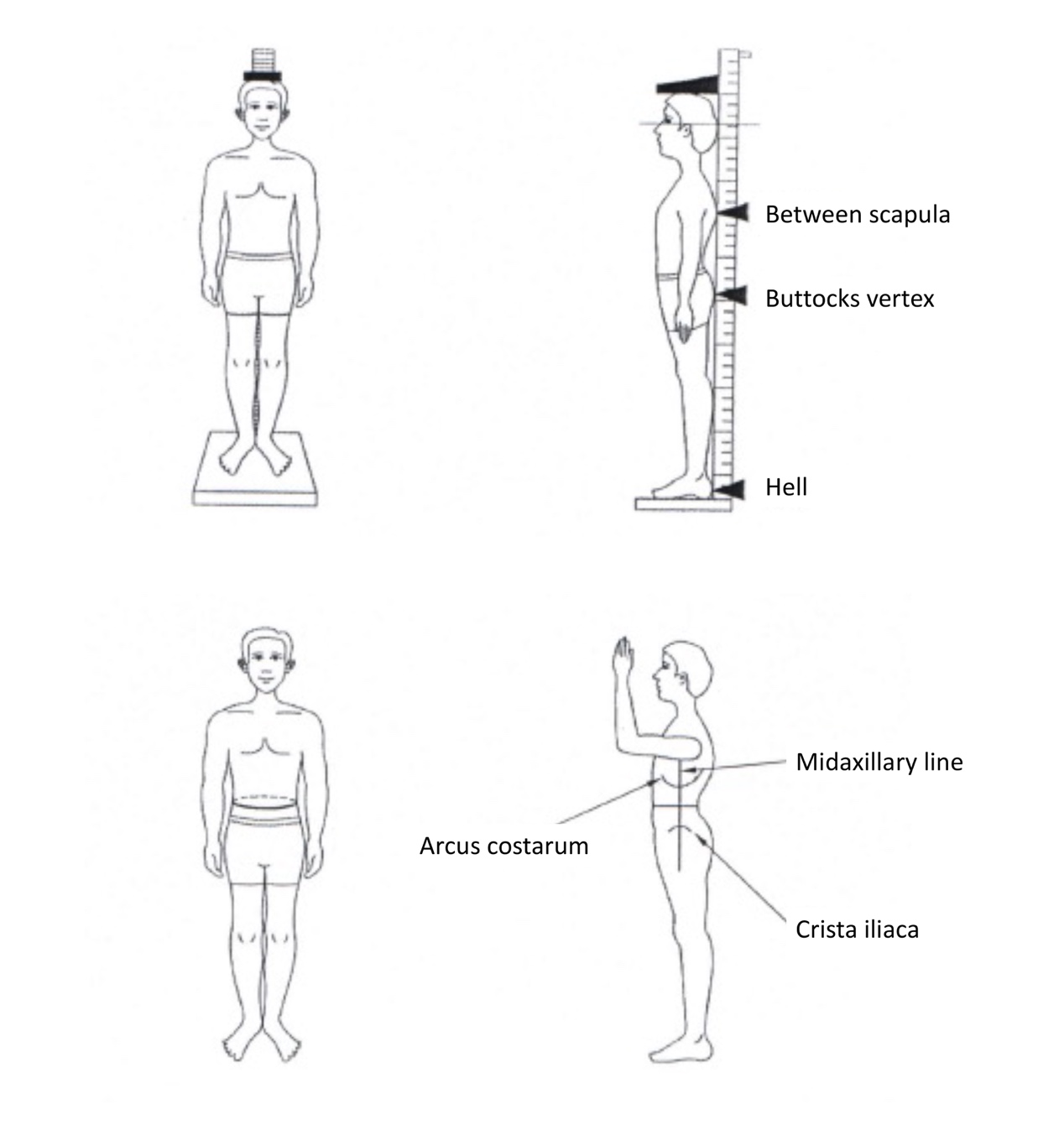


**Height and waistline measure position**

**Screening for Overweight and Obesity among School-age Children and Adolescents**

Health industry standard of People’s Republic of China WS/T 586-2018

Released by The Central People’s Government of the People’s Republic of China

Measure: Kg/m^2^

| Age (y) | Boy | | Girl | |
| --- | --- | --- | --- | --- |
|  | Overweight | Obesity | Overweight | Obesity |
| 6.0~ | 16.4 | 17.7 | 16.2 | 17.5 |
| 6.5~ | 16.7 | 18.1 | 16.5 | 18.0 |
| 7.0~ | 17.0 | 18.7 | 16.8 | 18.5 |
| 7.5~ | 17.4 | 19.2 | 17.2 | 19.0 |
| 8.0~ | 17.8 | 19.7 | 17.6 | 19.4 |
| 8.5~ | 18.1 | 20.3 | 18.1 | 19.9 |
| 9.0~ | 18.5 | 20.8 | 18.5 | 20.4 |
| 9.5~ | 18.9 | 21.4 | 19.0 | 21.0 |
| 10.0~ | 19.2 | 21.9 | 19.5 | 21.5 |
| 10.5~ | 19.6 | 22.5 | 20.0 | 22.1 |
| 11.0~ | 19.9 | 23.0 | 20.5 | 22.7 |
| 11.5~ | 20.3 | 23.6 | 21.1 | 23.3 |
| 12.0~ | 20.7 | 24.1 | 21.5 | 23.9 |
| 12.5~ | 21.0 | 24.7 | 21.9 | 24.5 |
| 13.0~ | 21.4 | 25.2 | 22.2 | 25.0 |
| 13.5~ | 21.9 | 25.7 | 22.6 | 25.6 |
| 14.0~ | 22.3 | 26.1 | 22.8 | 25.9 |
| 14.5~ | 22.6 | 26.4 | 23.0 | 26.3 |
| 15.0~ | 22.9 | 26.6 | 23.2 | 26.6 |
| 15.5~ | 23.1 | 26.9 | 23.4 | 26.9 |
| 16.0~ | 23.3 | 27.1 | 23.6 | 27.1 |
| 16.5~ | 23.5 | 27.4 | 23.7 | 27.4 |
| 17.0~ | 23.7 | 27.6 | 23.8 | 27.6 |
| 17.5~ | 23.8 | 27.8 | 23.9 | 27.8 |
| 18.0~ | 24.0 | 28.0 | 24.0 | 28.0 |

**Results**

**eTable 1 The Comparison between Survey Participants and Non-survey Participants Comparison**

| Item | Survey Participants  (N = 426) | Non-Survey participants  (N = 462) | *t/χ2* | *P* |
| --- | --- | --- | --- | --- |
| Age | 10.53 ± 1.68 | 10.39 ± 1.66 | 1.290 | .197 |
| Gender |  |  | 0.266 | .606 |
| Boy | 149 | 154 |  |  |
| Girl | 277 | 308 |  |  |
| BMIZ | 0.38 ± 1.18 | 0.42 ± 1.14 | -0.598 | .550 |
| WHtR | 0.45 ± 0.058 | 0.44 ± 0.059 | 0.518 | .604 |

**eTable 2 Factor Loadings of Items**

| Factor | Indicator | Estimate | SE | Z | *P* | Stand. Estimate |
| --- | --- | --- | --- | --- | --- | --- |
| Food Addiction | F1 | 0.552 | 0.0456 | 12.10 | < .001 | 0.554 |
|  | F2 | 0.593 | 0.0483 | 12.28 | < .001 | 0.561 |
|  | F3 | 0.260 | 0.0243 | 10.68 | < .001 | 0.498 |
|  | F4 | 0.553 | 0.0509 | 10.88 | < .001 | 0.507 |
|  | F5 | 0.487 | 0.0481 | 10.13 | < .001 | 0.475 |
|  | F6 | 0.459 | 0.0428 | 10.74 | < .001 | 0.500 |
|  | F7 | 0.448 | 0.0393 | 11.40 | < .001 | 0.526 |
|  | F8 | 0.372 | 0.0328 | 11.33 | < .001 | 0.524 |
|  | F9 | 0.579 | 0.0423 | 13.68 | < .001 | 0.613 |
|  | F10 | 0.285 | 0.0479 | 5.94 | < .001 | 0.290 |
|  | F11 | 0.573 | 0.0386 | 14.82 | < .001 | 0.653 |
|  | F12 | 0.557 | 0.0415 | 13.42 | < .001 | 0.605 |
|  | F13 | 0.555 | 0.0369 | 15.05 | < .001 | 0.662 |
|  | F14 | 0.252 | 0.0232 | 10.85 | < .001 | 0.505 |
|  | F15 | 0.603 | 0.0422 | 14.29 | < .001 | 0.634 |
|  | F16 | 0.459 | 0.0450 | 10.20 | < .001 | 0.478 |
|  | F17 | 0.479 | 0.0386 | 12.39 | < .001 | 0.565 |
|  | F18 | 0.395 | 0.0318 | 12.41 | < .001 | 0.566 |
|  | F19 | 0.485 | 0.0326 | 14.89 | < .001 | 0.656 |
|  | F20 | 0.290 | 0.0431 | 6.73 | < .001 | 0.327 |
|  | F21 | 0.194 | 0.0242 | 8.01 | < .001 | 0.385 |
|  | F22 | 0.360 | 0.0332 | 10.84 | < .001 | 0.504 |
|  | F23 | 0.649 | 0.0435 | 14.90 | < .001 | 0.656 |
|  | F24 | 0.314 | 0.0362 | 8.68 | < .001 | 0.414 |
|  | F25 | 0.685 | 0.0494 | 13.87 | < .001 | 0.621 |
|  | F26 | 0.565 | 0.0415 | 13.62 | < .001 | 0.610 |
|  | F27 | 0.288 | 0.0247 | 11.68 | < .001 | 0.538 |
|  | F28 | 0.495 | 0.0394 | 12.55 | < .001 | 0.571 |
|  | F29 | 0.373 | 0.0287 | 13.00 | < .001 | 0.588 |
|  | F30 | 0.500 | 0.0356 | 14.02 | < .001 | 0.625 |
|  | F31 | 0.577 | 0.0424 | 13.61 | < .001 | 0.612 |
|  | F32 | 0.653 | 0.0456 | 14.34 | < .001 | 0.638 |
|  | F33 | 0.407 | 0.0315 | 12.91 | < .001 | 0.585 |
|  | F34 | 0.311 | 0.0305 | 10.17 | < .001 | 0.478 |
|  | F35 | 0.773 | 0.0630 | 12.27 | < .001 | 0.561 |

**eTable 3 The Analysis of Food Addiction Scores in Diffe**rent Weight Groups (N = 426)

| Index | *N* | Median  (P25, P75) | Mean (deviation) | Z/χ2 | *P* |
| --- | --- | --- | --- | --- | --- |
| BMIZ |  |  |  | 52.460 | <0.001 |
| BMIZ ≤ 1 | 312 | 0.37 (0.17, 0.66) | 0.49 (0.42) |  |  |
| 1 < BMIZ ≤ 2 | 65 | 0.63 (0.40, 1.10) | 0.73 (0.46) |  |  |
| BMIZ > 2 | 49 | 0.94 (0.60, 1.44) | 1.03 (0.61) |  |  |
| WHtR |  |  |  | -6.902 | <0.001 |
| WHtR_boy_ < 0.48,  WHtR_girl_ < 0.46 | 307 | 0.37 (0.17, 0.66) | 0.48 (0.41) |  |  |
| WHtR_boy_ ≥ 0.48,  WHtR_girl_ ≥ 0.46 | 119 | 0.71 (0.43, 1.20) | 0.86 (0.56) |  |  |

Note: BMIZ, Body Mass Index Z score ; WHtR, Waist-height Ration.

**Appendix**

儿童版耶鲁食物成瘾量表2.0

Yale Food Addiction Scale Version 2.0 for Children

指导语：我们想了解你对于食物和吃的感受。以下问题的答案没有对错之分，因为每一个人的感受都是不同的。

我们只是想了解你对下列的感受，如：
-甜食例如冰淇淋、巧克力制品、甜甜圈、曲奇饼干、蛋糕、糖果、山楂条等
-咸味零食例如薯条、豆干、火腿肠、辣条、虾条、锅巴等
-高脂食物例如牛排、培根、汉堡包、奶酪棒、披萨和炸鸡等
-含糖饮料例如汽水、柠檬水、奶茶、和甜味乳饮料等
当提及“某些食物”，我们需要你想到类似上述食品的任何一种，句子中的“某些食物”就是指你最喜爱的食品。

Instruction

We want to know how kids feel about food and eating. There are no right or wrong answers. Every kid is different.

We just want to know how you feel about “junk foods” like:

- Sweets like ice cream, chocolate, doughnuts, cookies, cake, candy

- Salty snacks like chips, pretzels, and crackers

- Fatty foods like steak, bacon, hamburgers, cheeseburgers, pizza, and French fries

- Sugary drinks like soda pop, lemonade, sports drinks, and energy drinks

When we say “CERTAIN FOODS” we want you to think of ANY “junk food” like those above.

| 在过去一年（12个月）  IN THE LAST YEAR (PAST 12 MONTHS) | 从未  Never  0 | 很少  Rarely  1 | 有时  Some  times  2 | 经常  Very often  3 | 总是  Always  4 |
| --- | --- | --- | --- | --- | --- |
| 1.当我开始吃某些食物，我发现很难停下来。  1. When I started to eat certain foods, I found it hard to stop. |  |  |  |  |  |
| 2.尽管不饿，我还是会不停的吃某些食物。  2. I kept eating certain foods even though I was not hungry. |  |  |  |  |  |
| 3.我一直吃到胃疼或感到恶心为止。  3. I ate until my stomach hurt or I felt sick. |  |  |  |  |  |
| 4.我想过减少某些食物的摄入，但还是吃了。  4. I worried about cutting down on certain foods, but ate them anyway. |  |  |  |  |  |
| 5.我吃太多的时候，会有长时间的疲惫（狂吃之后会感到困、不想动弹、或反应变慢了）。  5. I spent a lot of time feeling tired from eating too much. |  |  |  |  |  |
| 6.我整天吃某些食物。  6. I ate certain foods all day long. |  |  |  |  |  |
| 7.如果我找不到我想要的食物，我会想尽办法得到它（比如：让朋友帮我拿，找一个自动售货机，趁别人不注意偷吃）。  7. If I could not find a food I wanted, I tried hard to get it. (examples: asked a friend to get it for me, found a vending machine, snuck food when people weren’t looking) |  |  |  |  |  |
| 8.我宁愿吃某些食物，也不愿做其他我喜欢的事情（比如：玩耍、和朋友们出去玩）。  8. I ate certain foods rather than do other things I like. (examples: play, hang out with friends) |  |  |  |  |  |
| 9.因为我吃太多，我和我的家人或朋友有过争执。  9. I had fights with my family or friends because I ate too much. |  |  |  |  |  |
| 10.我会避免去有某些食物的地方，因为我可能会吃太多（例如：聚会、朋友家）。  10. I avoided places that had certain foods, because I might eat too much. (examples: parties, friends’ houses) |  |  |  |  |  |
| 11.当我减少或停止吃某些食物时，我会感觉到生气、心烦或者难过。  11. When I cut down on or stopped eating certain foods, I felt angry, upset or sad. |  |  |  |  |  |
| 12.如果我因为没有吃某些食物而感到不舒服，我就会去吃这些食物来使自己感觉好一些。  12. If I felt sick because I hadn’t eaten certain foods, I would eat those foods to feel better. |  |  |  |  |  |
| 13.如果我因为没有吃某些食物而感到心烦，我就会去吃这些食物来使自己感觉好一些。  13. If I was upset because I hadn’t eaten certain foods, I would eat those foods to feel better. |  |  |  |  |  |
| 14.当我减少或停止吃某些食物，我会感觉不舒服。比如：我会头疼或很累。  14. When I cut down on or stopped eating certain foods, I felt sick. For example, my head hurt or I was really tired. |  |  |  |  |  |
| 15.当我减少或停止吃某些食物，我对它们的渴求更强烈。  15. When I cut down or stopped eating certain foods, I craved them a lot more. |  |  |  |  |  |
| 16.我吃东西的方式使我很不开心（对吃上瘾，想要停止这种吃东西的方式）。  16. The way I ate made me really unhappy. |  |  |  |  |  |
| 17.我吃东西的方式给我带来了一些问题（例如：在学校、和父母、朋友之间的问题）。  17. The way I ate caused me problems. (examples: problems at school, with parents, with friends). |  |  |  |  |  |
| 18.我吃太多之后会感觉很糟糕，以至于不（想）去做我喜欢的事情了（比如：玩耍，和朋友出去玩）。  18. I ate so much that I felt bad afterwards. I felt so bad that I did not do things I like. (examples: play, hang out with friends) |  |  |  |  |  |
| 19.我吃的太多，而没有去做其他重要的事情（比如：家庭作业、家务活）。  19. I ate so much that I did not do other important things. (examples: homework, chores) |  |  |  |  |  |
| 20.我避开不能得到我想要的食物的地方。  20. I avoided places where I could not get the foods I wanted. |  |  |  |  |  |
| 21.我避免和其他孩子出去玩，因为他们认为我吃的太多了。  21. I avoided hanging out with other kids because they thought I ate too much. |  |  |  |  |  |
| 22.我一直吃的太多，尽管这让我感觉难过、紧张或内疚。  22. I kept eating too much even though it made me feel sad, nervous, or guilty. |  |  |  |  |  |
| 23.尽管这不健康，我还是会吃很多。  23. I kept eating too much even though it made me unhealthy. |  |  |  |  |  |
| 24.当我吃相同分量的食物时，它并不能像之前那样让我感觉良好了（例如：感觉开心、平静、放松）。  24. When I ate the same amount of food, it didn’t make me feel as good as it used to. (examples: feel happy, calm, relaxed) |  |  |  |  |  |
| 25.我真的想要减少或停止吃某种食物，但是我就是做不到。  25. I really wanted to cut down on or stop eating certain kinds of foods, but I just couldn’t. |  |  |  |  |  |
| 26.我需要吃更多来获得我从吃中想要的良好感觉（比如：感到快乐、平静或放松）。  26. I needed to eat more to get the good feelings I wanted from eating. (examples: feel happy, calm or relaxed) |  |  |  |  |  |
| 27.因为吃的太多，我在学校的表现不好（对食物有强烈渴求，以至于对学校布置的任务不能集中注意力／暴饮暴食之后感到疲惫，而不能很好的完成作业）。  27. I didn’t do well at school because I was eating too much. |  |  |  |  |  |
| 28.我一直吃某些食物，尽管我知道这是危险的（比如：即使我有糖尿病我也会吃甜食；即使我知道这些食物不健康，但还是会吃）。  28. I kept eating certain foods even though I knew it was dangerous. (example: eating sweets even though I had diabetes) |  |  |  |  |  |
| 29.我有吃某些食物的强烈欲望，以至于我根本不能想其他事情。  29. I had such strong urges to eat certain foods that I couldn’t think of anything else. |  |  |  |  |  |
| 30.我非常渴求吃某些食物，以至于我觉得我必须马上吃到它们。  30. I was craving certain foods so much that I felt like I had to eat them right away. |  |  |  |  |  |
| 31.我试着少吃某些食物，但没有用。  31. I tried to cut down on certain foods, but it didn’t work. |  |  |  |  |  |
| 32.我尝试不吃某些食物，但失败了。  32. I tried and failed to stop eating certain foods. |  |  |  |  |  |
| 33.吃东西很让我分心，这可能会让我受伤（比如：过马路时边走边吃）。  33. I was so distracted by eating that I could have been hurt. (e.g., crossing the street) |  |  |  |  |  |
| 34.想着食物很让我分心，这可能会让我受伤（比如：过马路时在想着某些吃的）。  34. I was so distracted by thinking about food that I could have been hurt. (e.g., crossing the street) |  |  |  |  |  |
| 35.我的朋友或家人担心我吃得太多。  35. My friends or family were worried that I ate too much. |  |  |  |  |  |

选项解释：

- 从不/从未：从来没有过；
- 极少/很少/偶尔：仅有过一两次，或有过这种情况，但发生次数很少；
- 有时：有时候如此；
- 经常：大部分情况如此；
- 总是：一直如此。
